# Chemical Composition, Antimicrobial Properties of *Siparuna guianensis* Essential Oil and a Molecular Docking and Dynamics Molecular Study of its Major Chemical Constituent

**DOI:** 10.3390/molecules25173852

**Published:** 2020-08-25

**Authors:** Mozaniel Santana de Oliveira, Jorddy Neves da Cruz, Wanessa Almeida da Costa, Sebastião Gomes Silva, Mileide da Paz Brito, Sílvio Augusto Fernandes de Menezes, Antônio Maia de Jesus Chaves Neto, Eloisa Helena de Aguiar Andrade, Raul Nunes de Carvalho Junior

**Affiliations:** 1 Program of Post-Graduation in Food Science and Technology, LABEX/FEA (Faculty of Food Engineering), Federal University of Para, Rua Augusto Corrêa S/N, Guamá, Belém PA 66075-900, Brazil; 2 Adolpho DuckeLaboratory, Botany Coordination, Museu Paraense Emílio Goeldi, Av. Perimetral, 1900, Terra Firme, Belém PA 66077-830, Brazil; jorddynevescruz@gmail.com (J.N.d.C.); professebastiao@yahoo.com.br (S.G.S.); eloisandrade@ufpa.br (E.H.d.A.A.); 3 Program of Post-Graduation in Natural Resources Engineering, Federal University of Para, Rua Augusto Corrêa S/N, Guamá, Belém PA 66075-900, Brazil; wanessa.almeida712@yahoo.com (W.A.d.C.); amchaves@ufpa.br (A.M.d.J.C.N.); 4 The University Center of Pará State - Av. Governador José Malcher, 1963 - Nazaré, Belém PA 66075-900, Brazil; mileidepb@yahoo.com.br (M.d.P.B.); menezesperio@gmail.com (S.A.F.d.M.); 5 Laboratory of Preparation and Computation of Nanomaterials, Federal University of Para, Rua Augusto Corrêa S/N, Guamá, Belém PA 66075-900, Brazil; 6 Program of Post-Graduation in Chemistry, Federal University of Para, Rua Augusto Corrêa S/N, Guamá, Belém PA 66075-900, Brazil

**Keywords:** Amazon, natural products, Capitiú, biomolecules, volatile compounds

## Abstract

The essential oil of *Siparuna guianensis* was obtained by hydrodistillation. The identification of the chemical compounds was performed by gas chromatography coupled with mass spectrometry (GC/MS). Antimicrobial activity was investigated for four microorganisms: *Streptococcus mutans* (ATCC 3440), *Enterococcus faecalis* (ATCC 4083), *Escherichia coli* (ATCC 25922), and *Candida albicans* (ATCC-10231). The studies of doping and molecular dynamics were performed with the molecule that presented the highest concentration of drug–target proteins, 1IYL (*C. albicans*), 1C14 (*E. coli*), 2WE5 (*E. faecalis*), and 4TQX (*S. mutans*). The main compounds identified were: Curzerene (7.1%), γ-Elemene (7.04%), Germacrene D (7.61%), *trans*-β-Elemenone (11.78%), and Atractylone (18.65%). Gram positive bacteria and fungi were the most susceptible to the effects of the essential oil. The results obtained in the simulation showed that the major compound atractylone interacts with the catalytic sites of the target proteins, forming energetically favourable systems and remaining stable during the period of molecular dynamics.

## 1. Introduction

Fungi and bacteria can cause various pathologies in humans. Leprosy [1], tuberculosis [2], bacterial dysentery [3], gonorrhea [4], urinary tract infection, endocarditis [5,6], onychomycosis [7], mucormycosis [8], and candidiasis [9] are examples of diseases that these microorganisms can cause. In some cases, the symbiosis between bacteria and fungi increases the virulence of bacteria, because fungi such as *C. albicans* elevates the production of exopolysaccharides, which can become an ideal shelter for *S. mutans*, thus making it difficult to control this microorganism [10]. Another important factor is the resistance that microorganisms are developing to traditional antibiotics, since this poses a threat to public health and is associated with high rates of morbidity and mortality [11]. In this sense, natural products, more specifically essential oils, can become a viable alternative for the control of fungi and bacteria [12,13].

The plants that produce essential oils (EOs) have been an object of study for years, since their EOs present varied biological activities [14], such as cytotoxic, antimicrobial, antioxidant [15], anti-inflammatory, anti-proliferative [16,17,18], antibacterial, antifungal [19,20,21,22,23,24], antiviral [25,26], anticonvulsant [27,28], analgesic [29], and neuroprotective properties [30]. As a result, they are increasingly attracting the attention of many industry segments [31]. Essential oils consist of a complex mixture of volatile organic substances, often involving 50, 100, or even more isolated components, and that contain chemical groups such as hydrocarbons, alcohols, aldehydes, ketones, acids, and esters [32].

*Siparuna guianensis* was the first *Siparuna* species described and illustrated by Aublet [33]. This plant is present from Nicaragua to Paraguay, and in Brazil, this species is known by several names, such as *negramina*, *folha*-*santa*, *marinheiro*, *capitiú*, *mata*-*cachorro*, *catingoso*, *limão*-*bravo*, *cicatrizante*-*das*-*guianas*, *catingueira*-*de*-*paca*, and *fedegoso*. In many countries of America, leaves of *S. guianensis* are widely used as a drink to combat stomach pains [34] and this activity may be related to the compounds present in its essential oil [35,36]. In addition, there is little research that reports on the antimicrobial activity of *Siparuna guianensis* essential oil, [37] including a chemotype found in Tocantins, Brazil. In this context, the objective of this work was to evaluate the chemical composition, antimicrobial activity and simulate the mechanisms of interaction of the major chemical constituent present in the essential oil of *Siparuna guianensis*, using doping techniques and molecular dynamics.

## 2. Results and Discussion

### 2.1. Yield and Chemical Composition

**Figure 1:**
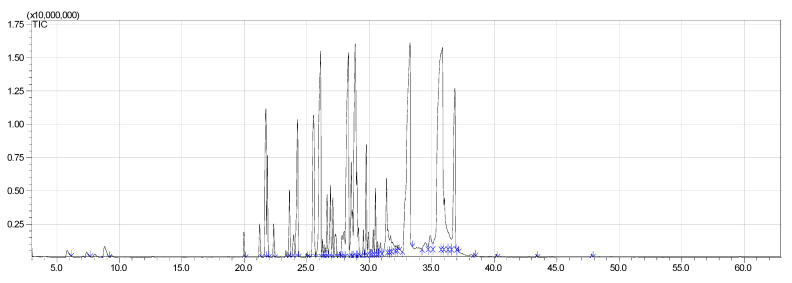
Ion chromatogram relative to the chemical composition *S. guianensis* essential oil.

The moisture content of the *S. guianensis* sample was 13.58% and the volume of essential oil obtained in the hydrodistillation was 0.5 mL, with a yield of 1.42% (db). Regarding the chemical profile of the essential oil of *S.guianensis*, 51 compounds were identified, the most important being *trans*-β-Elemenone (11.78%) and Atractylone (18.65%), followed by δ-Elemene (5.38%), β-Elemene (3.13%), β- Yerangene (4.14%), γ-Elemene (7.04%), Germacrene D (7.61%), Curzerene (7.1%), and Germacrone (5.26%) (See Table 1). In Figure 1, the ion chromatogram relative to the chemical composition can be observed. Cicció and Gómez [38] analyzed the essential oil of *Siparuna thecaphora* obtained by hydrodistillation and the compounds obtained in the highest concentrations were germacrene D (32.7%), α-pinene (16.3%), β-pinene (13.8%), and e β-caryophyllene (4.1%). In a similar study with *Siparuna guianensis* [39], they found myrcene (28.74%) [40], β-myrcene (13.14%), and the sesquiterpenes germacrene-D (8.68%) and bicyclogermacrene (16.71%).

In a study related to the chemical composition of *S. guianensis* essential oil from southeastern Brazil, they obtained high concentrations of capric acid (46.6%) and 2-undecane (31.7%) [41]. These compounds were not identified in other studies such as Zoghbi et al. [42], who analysed the chemical composition of *S. guianensis* essential oil, collected in various cities of Northern Brazil and identified epi-α-bisabolol (25.1%) and spathulenol (15.7%) in Moju (PA), spathulenol (22.0%), selin-11-en-4α-ol (19.4%), β-eudesmol (10.0%), and elemol (10.0%) in leaves collected in Rio Branco (AC), and germacrone (23.2%), germacrene D (10.9%), bicyclogermacrene (8.6%), germacrene B (8.0%) and atractylon (31.4%) in Belém (PA). The results found in other studies [43,44,45] show that the chemical composition of the essential oil of *S. guianensis* varies according to the seasonality and site of collection.

### 2.2. Antimicrobial Activity

The antimicrobial activity analysed by the diffusion method can be observed in Table 2. The microorganisms presented mean inhibition halos of 11 ± 0.12 (mm), 12 ± 0.57(mm), 11 ± 0.31(mm), and 12.5 ± 0.98 (mm) for Gram-positive *Streptococcus mutans* (ATCC 3440), Gram-positive *Enterococcus faecalis* (ATCC-4083), Gram-negative *Escherichia coli* (ATCC 25922), and *Candida albicans* (ATCC-10231), respectively. *Streptococcus mutans* (ATCC-3440) and *Candida albicans* (ATCC- 10231) were the most sensitive to the effects of essential oils, with a minimum inhibitory concentration of 125 μL/mL, whereas the bacterium *Enterococcus faecalis* (ATCC-4083) [36] demonstrated that the essential oil of *S. guianensis* exerts an inhibitory effect on fungi, and on Gram-negative and Gram-positive bacteria.

In general, Gram-positive bacteria were the most sensitive to the effects of essential oil (EO), and this may be related to the fact that Gram-positive bacteria are more susceptible to the effects of volatile components compared to the Gram-negative ones [48]. In the case of fungi, EOs can be a viable alternative in the fight against the infection caused by *Candida* [24,49]. These biological effects can be related to the presence of chemically active compounds such as γ-elemene, curzerene, germacrene D, β-elemenone, and atractylon, as there are reports in the literature that corroborate this thesis [50,51,52].

## 3. Interaction Mechanism

### 3.1. Molecular Binding Mode

From our molecular docking results, it can be suggested that the ligand interacts favourably with the target proteins. In Table 3, the results of the MolDock score for each complex formed are presented.

**Figure 2:**
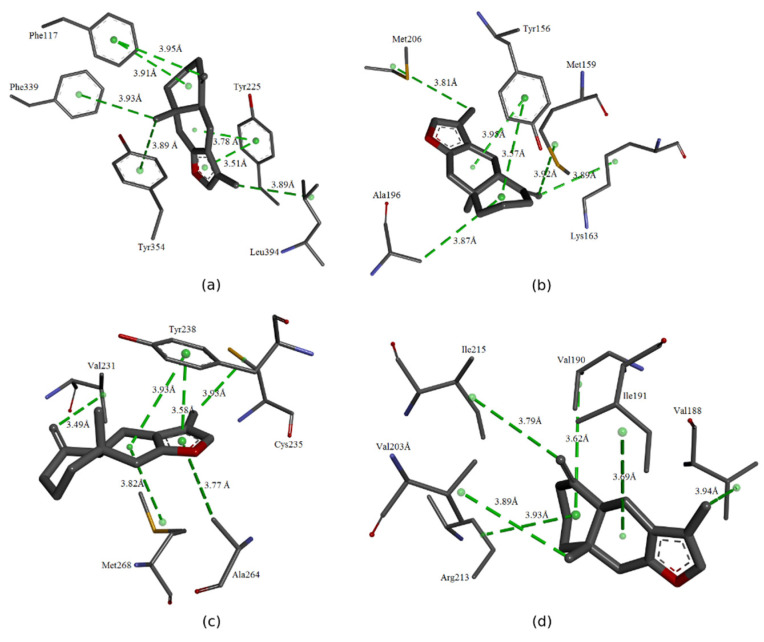
Molecular interactions between ligand-receptor. (**a**) Molecular binding of atractylon with the protein N-myristoyltransferase of the microorganism *C. Albicans*, (**b**) Molecular binding of atractylon with the protein Enoyl reductase of the microorganism *E. Coli*, (**c**) Molecular binding of atractylon with the protein Carbamate kinase of the microorganism *E. faecalis*, and (**d**) Molecular binding of atractylon with the protein Sortase A of the microorganism *S. mutans*.

The interactions between atractylon, the primary compound from the essential oil isolated from the leaves of *Siparuna guianensis*, and the catalytic site of the enzymes were analysed. The interactions that were formed are visualized in Figure 2.

In Figure 2A, it is possible to observe that the ligand performed several hydrophobic interactions with different residues of the catalytic site of N-myristoyltransferase (*C. albicans*). With the Tyr225 residue, two interactions were established, one of the pi-pi types and the other of the pi-alkyl type. Residues Phe339 and Tyr354 had pi-alkyl-type interactions with the ligand, whereas Leu394 established alkyl interactions. Phe117 was also able to form two interactions, both of the pi-alkyl type.

With Enoyl reductase residues (*E. coli*), atractylon established six hydrophobic interactions. Four of these interactions were of the alkyl type with the following residues: Met206, Met159, Lys163, and Ala196. In addition, two additional pi-alkyl-type interactions with Tyr156 were formed.

The interaction of the ligand with the binding pocket of the enzyme Carbamate kinase (*E. faecalis*) can be seen in Figure 2C. With residues Val231, Cys235, and Met268, hydrophobic interactions of the alkyl type were formed. With Tyr238, two interactions were established, one of the pi-alkyl type and one of the pi-pi type. With Ala264, an interaction of the same type was formed.

All interactions formed with the residues of Sortase A (*S. mutans*) were of hydrophobic and alkyl types. These interactions were established with the following residues: Ile215, Val190, Ile191, Val188, Arg213, and Val2013.

### 3.2. Analysis of Complexes Stability

**Figure 3:**
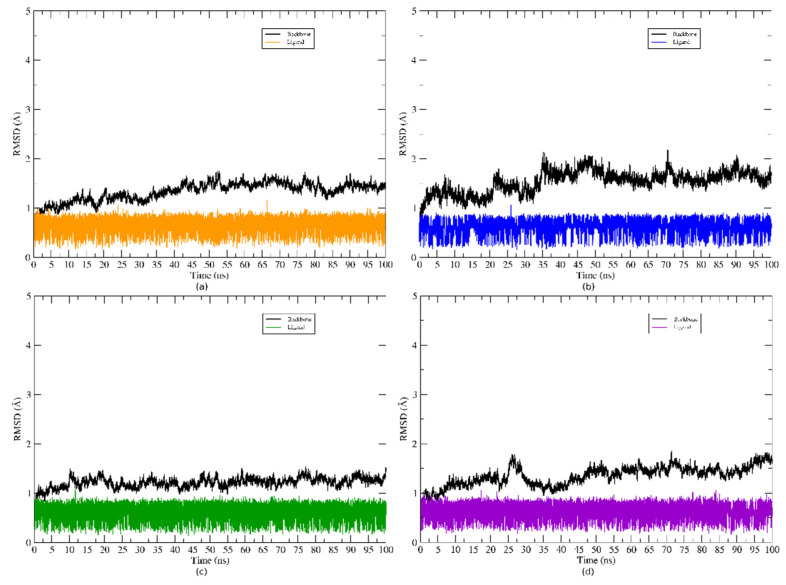
RMSD of systems for 100 ns of MD simulations. The black colour was used to colour the backbone of all proteins, whereas various colours were used for the ligand RMSD. (**a**) RMSD plot of the atractylon/N-myristoyltransferase system (*C. albicans*), (**b**) RMSD plot of the atractylon/Enoyl reductase system (*E. coli*), (**c**) RMSD plot of the atractylon/Carbamate kinase system (*E. faecalis*), and (**d**) RMSD plot of the atractylon/Sortase A system (*S. mutans*).

The complexes obtained by molecular docking were used as a starting point for molecular dynamics simulations. The root mean square deviation (RMSD) graphs were plotted in relation to the lowest energy structure obtained for the systems, after the execution of the protocol of energy minimization, heating, and equilibrium. To plot the RMSD of the proteins’ backbone, their Cα atoms were used and to plot the RMSD of the ligands, their heavy atoms were used. The correspondent graphs can be seen in Figure 3.

For the systems formed with the target proteins of *C. albicans*, *E. coli*, *E. faecalis* and *S. mutans*, the mean RMSD obtained for the ligand was 0.65 Å, 0.62 Å, 0.66 Å, and 0.64 Å, respectively. Thus, it is possible to infer that during the simulations, the inhibitor remained stable at the binding site of the different targets during molecular dynamics.

The target proteins showed small conformational changes as can be observed in the RMSD plots. These changes resulted from the accommodation of the ligands at their respective binding sites.

The fluctuations observed in the RMSD for the proteins backbone may be the result of the accommodation of the ligand at the active site. The mean values for the RMSD were relatively low. These values for the backbone of the target proteins of *C. albicans, E. coli, E. faecalis*, and *S. mutans* were 1.63 Å, 1.53 Å, 1.44 Å, and 1.65 Å, respectively.

### 3.3. Free Energy Calculations Using MM/GBSA Approach

For each complex, the values of affinity energy (ΔG_MM-GBSA_), in addition to the values of the energetic contributions involved in the ligand-receptor interaction were obtained. The energy contributions obtained were as follows: van der Waals (ΔE_vdW_), polar (ΔG_GB_), non-polar (ΔG_NP_), and the electrostatic interactions energies (ΔE_ele_) (Table 4).

In all systems, the free energy values demonstrated that atractylon is capable of inhibiting enzymatic activity. The contributions of Van der Waals were the most responsible for the interaction of the ligand with the molecular targets. Moreover, the electrostatic and nonpolar contributions were favourable for the maintenance of the complexes.

## 4. Materials and Methods

### 4.1. Preparation and Characterization of the Siparuna guianensis Sample

**Figure 4:**
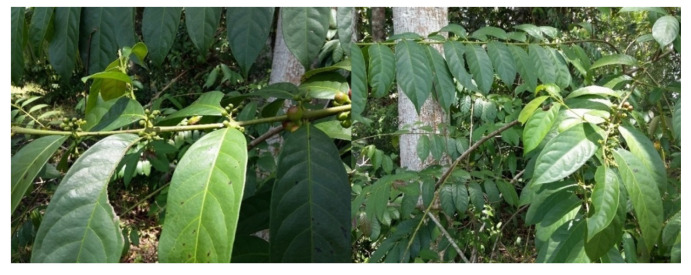
Leaves of *Siparuna guianensis* before collection.

The *Siparuna guianensis* sample was obtained in the herbarium of the Museu Paraense Emilio Goeld (Eastern Amazon), on 09/09/2016. The geographical coordinates of the collection site were S01°27′04.3″ and W048°26′38.3″, with a relative humidity of 64.9% and temperature of 26.5 °C. The samples were identified by Dr. Antonio Elielson Sousa da Rocha, and then, its registration number was incorporated in the Emílio Goeldi Museum Herbarium, located in the city of Belém, Pará, Brazil, under the v-oucher *MG-165435*. Before the extraction process, the sample was dried and ground and then the moisture content was determined by infrared moisture analyser. The images of the leaves of *S. guianensis* can be observed in Figure 4.

#### 4.1.1. Botanical Information of the Sample

Capitiú (*Siparuna guianensis*) belongs to the *Siparunaceae* Family. It is a shrub about three meters high, the immature fruits were greenish, and the ripe ones were greenish and purplish, with short pedunculated axillary racemes, and opposite, elliptic, and lanceolate leaves. This plant releases a characteristic odour of fish. Leaf samples were identified and deposited in the medicinal plants herbarium of the Museu Paraense Emílio Goeldi.

#### 4.1.2. Extraction Procedure: Hydrodistillation

After the drying process, the leaves of *S. guianensis* were submitted to hydrodistillation using a Clevenger-type extractor. For the extraction process, 40 g of the sample was used, for 10,800 s at 100 °C. After this procedure, anhydrous sodium sulphate (Na_2_SO_4_) was added and the essential oil was centrifuged to be moisture-free. The essential oil yield was calculated in dry basis (db).

### 4.2. Analysis of Volatile Compounds

The chemical composition of the essential oils was evaluated by gas chromatography/mass spectrometry (GC/MS) according to the methodologies by [53,54], using a Shimadzu QP-2010 plus system under the following conditions: silica capillary column Rtx-5MS (30 m × 0.25 mm, 0.25 μm film thickness); program temperature of 60–240 °C at 3 °C/min; injector temperature of 250 °C; helium as carrier gas (linear velocity of 32 cm/s, measured at 100 °C); splitless injection (1 μL of a 2:1000 hexane solution). Ionization was obtained by electronic impact technique at 70 eV, and the temperature of the ion source and other parts was set at 200 °C. The quantification of volatile compounds was determined by gas chromatography with a flame ionization detector (FID; Shimadzu, QP 2010 system-Kyoto, Japan) under the same conditions as gas chromatography coupled to mass spectrometry (GC-MS), except that hydrogen was used as the carrier gas. The retention index was calculated for all volatile constituents using a homologous series of n-alkanes (C_8_–C_20_), and were identified by comparing the mass spectra obtained experimentally and their retention indices to those found in literature [46,47].

### 4.3. Analysis of In vitro Antimicrobial Activity

In the microbiological assays, standard strains of *Streptococcus mutans* (ATCC 3440), *Enterococcus faecalis* (ATCC 4083), *Escherichia coli* (ATCC 25922), and *Candida albicans* (ATCC 10231) were used. All of them were purchased from the Osvaldo Cruz Foundation (FIOCRUZ-Rio de Janeiro, Brazil), belonging to the base of standards of the Laboratory of Microbiological Quality Control of Medicines of the University Center of Pará-CESUPA.

The inoculum of each microorganism was obtained from a microbial suspension of fresh culture (maximum 24 h) in saline solution 0.85% (*m*/*V*), by comparing the inoculum turbidity with the MacFarland scale, equivalent to a concentration of 1.5 × 108 UFC/mL [55] in a turbidimeter (Grant bio, Model: DEN-1Shepreth, Cambridge, UK).

The culture medium used for the disk diffusion test was soybean casein agar (SCA) and brain Heart Infusion (BHI) broth containing 0.2% polysorbate 80 (*m*/*V*). 5% (*v*/*v*) of sheep blood was added for the analysis of strains of *Streptococcus mutans* (ATCC 3440) and *Enterococcus faecalis* (ATCC 4083).

### 4.4. Evaluation of the Sample Sensitivity by the Disk Diffusion Method

Briefly, 10 mL of Soybean Casein Agar (15 × 100 mm) was poured into a Petri dish. The microorganism (106 CFU/mL) was then inoculated with the aid of a sterile swab and paper discs (6 and 8 m) impregnated with 10 μL of oil. Positive and negative control were added onto the medium. The plates were incubated at 30 ± 5 °C/24 h in an aerobic environment [22,55,56]. The analysis was performed in triplicate. After the incubation period, the plates were revealed with triphenyltetrazolic chloride at 7 mg/mL in bacteriological agar at 1% (*w*/*v*). The halos were measured using a pachymeter (mm) and evaluated by a descriptive analysis.

### 4.5. Determination of Minimum Inhibitory Concentration (MIC)

The Minimum inhibitory concentration MIC was performed with the essential oil and was adapted from the micro dilution proposed by [55]. The test was performed on an Elisa^®^ plate, where a 100 μL sample aliquot was diluted (1:2 *v*/*v*) in BHI broth containing 106 CFU/mL until 10 consecutive dilutions, and then positive and negatives controls were added. Plates were incubated at 30 ± 5 °C/48 h. The test was performed in triplicate.

After incubation, plates were revealed with 1% (m/v) bacteriological broth containing 7 mg/mL triphenyltetrazolic chloride solution and incubated for further 30 min at 30 ± 5 °C for bacteria, and at 25 ± 5 °C for *C. albicans*. The maintenance of the red colour in the medium was interpreted as microbial growth.

### 4.6. Statistical Analysis

The statistical analysis was performed using the application MiniTab17-State College, Pennsylvania, USA, using the means and their respective standard deviations.

## 5. Molecular Docking and Dynamics Molecular Simulations

### 5.1. Molecular Docking

For the study of molecular docking, atractylone was selected, since it was the primary compound from the essential oil isolated from the leaves of *Siparuna guianensis*. Molecular docking was used to investigate the interaction between atractylone and essential proteins of *C. albicans*, *E. coli*, *E. faecalis*, and *S. mutans*. The proteins used as a molecular target are essential for the metabolic pathways of such microorganisms, in addition to being reported in the literature as targets for natural and synthetic products that combat these pathogens [57,58,59,60].

The chemical structure of atractylon, after being designed with GaussView 5.5 software - Wallingford, Connecticut United States, was optimized with B3LYP/6-31G* [61], using Gaussian 16 (Wallingford, CT, USA) [62]. To study the interaction mode of this molecule with target-proteins for drug action, the software Molegro Virtual Docker 6 (Århus, Denmark, https://molegrovirtualdocker.weebly.com/) was used [63,64]. The crystal structures of the proteins used as targets can be found in the Protein Data Bank (www.rcsb.org), from their ID: 1IYL (*C. albicans*) [57], 1C14 (*E. coli*) [58], 2WE5 (*E. faecalis*) [59], and 4TQX (*S. mutans*) [60]. The MolDock Score (GRID) function was used with a Grid resolution of 0.30 Å and radius of 7 Å, encompassing the entire crystallographic ligand binding cavity found in the PDB of each protein. The MolDock SE algorithm was used with number of runs equal to 10; 1500 max interactions, and max population size equal to 50. The maximum evaluation of 300 steps with neighbor distance factor equal to 1 and energy threshold equal to 100 were used during the molecular docking simulation. The RMSD limit for multiple cluster poses was set to < 1.00 Å.

### 5.2. Molecular Dynamics (MD) Simulation

The ligand parameters were constructed with the aid of the Antechamber module, using the General Amber Force Field (GAFF) [65]. The calculations to determine the atomic charges of the ligand were performed according to the restrained electrostatic potential (RESP) protocol using basis set Hartree–Fock level might with the functional 6-31G* [66]. To measure the protonation status of the amino acid residues of the receptors, the results obtained from the PROPKA program were used [67,68].

In the molecular dynamics simulations, the force field ff14SB [69] and the explicit water molecules described by the TIP3P model [70] were used. All systems were solvated in an octahedron periodic box, where a cutting radius of 12Å was applied in all directions from the solute. Finally, in each system, an adequate number of counter-ions were added to neutralize the charge.

The MD simulations were performed with the Amber 16 package [71,72,73]. Sander. MPI was used for the energy minimization steps, and pmemd. CUDA, for the heating, equilibrium and MD simulations.

The energy minimization of the systems occurred in three stages. In the first step, 1500 cycles were performed using steepest descent method and conjugate gradient algorithm, applying a harmonic force constant of 100 kcal/mol.Å^−2^ on the solute. In the second step, the harmonic force constant applied on the solute was 50 kcal/mol.Å^−2^ and further 500 cycles were performed using the steepest descent method and conjugate gradient algorithm. In the last step, the restrictions were removed, and 500 cycles were performed using the same protocol.

To raise the systems temperature from 0 to 300k, 800 ps of simulations were performed. The heating was carried out in three stages. In the first stage, the solute was restricted with a harmonic force constant of 50 kcal/mol.Å^−2^. Thus, only the solvent and the counter-ions get free to move. In the next two steps, the harmonic force constant was removed.

To balance the complexes, 2 ns of simulations with constant temperature and with no restrictions were performed. Then, for each complex, 100 ns of MD simulation were obtained with NVT ensemble.

The particle mesh Ewald method [74] was used for the calculation of electrostatic interactions, and the bonds involving hydrogen atoms were restricted with the SHAKE algorithm [75]. The temperature control was performed with the Langevin thermostat [76] within collision frequency of 2 ps^−1^.

### 5.3. Free Energy Calculations

The binding free energy was calculated using the molecular mechanics-generalized Born surface area (MM-GBSA) approach [77,78,79]. For the affinity energy calculation, 500 snapshots of the last 5 ns of the MD simulations trajectories were used.

The free energy was calculated according to the following equations:

(1) $$\Delta G_{\mathrm{bind}}\;=\;\Delta H\;-\;T\Delta S\;\approx \;\Delta E_{\mathrm{MM}}\;+\;\Delta G_{\mathrm{solv}}\;-\;T\Delta S\;$$

where ΔG_bind_ is the free energy of the complex, which is the result of the sum of the molecular mechanics energy (ΔE_MM_), the desolvation free energy (ΔG_solv_), and the entropy (−TΔS).

(2) $$\Delta E_{\mathrm{MM}}\;=\;\Delta E_{\mathrm{internal}}\;+\;\Delta E_{\mathrm{electrostatic}}\;+\;\Delta E_{\mathrm{vdW}}\;$$

The energy of molecular gas phase mechanics (ΔE_MM_) can be described by the sum of the internal energy contributions (ΔE_internal_), the sum of the connection, angle and dihedral energies, electrostatic contributions (ΔE_eletrostatic_), and van der Waals terms (ΔE_vdW_).

(3) $$\Delta \mathrm{Gsolv}\;=\;\Delta G_{\mathrm{GB}}\;+\;\Delta G_{\mathrm{nonpol}}\;$$

Desolvation free energy (ΔG_solv_) is the sum of the polar (ΔG_GB_) and non-polar (ΔG_nonpol_) contributions. The polar desolvation term was calculated using the implicit generalized born (GB) approaches.

## 6. Conclusions

The main compounds obtained in the essential oil of *Siparuna guianensis* were Atractylone (18.65%), *trans*-β-Elemenone (11.78%), Germacrene D (7.61%), Curzerene (7.1%), γ-Elemene (7.04%) and, followed by δ-Elemene (5.38%), Germacrone (5.26%), β- Yerangene (4.14%), and β-Cubebene (3.34%). The bacterium most sensitive to the effect of the essential oil was *Streptococcus mutans* followed by the fungus *Candida albicans.* Both microorganisms had the same MIC value (125 μL / mL). In our results, it is evidenced that atractylon interacts with all catalytic sites of the proteins and may be an inhibitor. The energy contributions observed were the electrostatic interactions energies (ΔE_ele_), and of the van der Waals (ΔEvdW), polar (ΔGGB), and nonpolar (ΔGNP) types.

## Figures and Tables

**Table 1:** Chemical compounds identified in the essential oil of *S. guianensis* and their relative concentrations (%).

Rt	RI_C_	RI_L_	Compound	PubChem CID/SID or Chemical Structure	Concentration (%)
5.817	933	932 ^a^	α-pinene	6654	0.33
7.034	978	974 ^a^	β-pinene	14896	0.04
7.375	989	988 ^a^	myrcene	31253	0.22
7.465	1008	1002 ^a^	α-phellandrene	7460	0.15
8.808	1030	1025 ^a^	sylvestrene	12304570	0.51
8.998	1044	1044 ^a^	(*E*)-β-ocimene	5281553	0.07
19.958	1292	1293 ^a^	undecan-2-one	8163	0.38
21.842	1331	1335 ^a^	δ-elemene	12309449	5.38
22.342	1345	1345 ^a^	α-cubebene	86609	0.48
23.317	1367	1373 ^a^	α-ylangene	442409	0.12
23.608	1373	1374 ^a^	α-copaene	442355	1.1
23.879	1381	1387 ^a^	β-bourbonene	62566	0.52
23.942	1383	1389 ^a^	β-elemene	6918391	0.55
24.258	1386	1387 ^a^	β-Cubebene	93081	3.34
24.654	1392	1402 ^a^	α-funebrene	6552024	0.03
24.967	1404	1409 ^a^	α-gurjunene	15560276	0.06
25.158	1408	1417 ^a^	(*E*)-caryophyllene	5281515	0.03
25.525	1417	1419 ^a^	β-ylangene	519779	4.14
26.1	1430	1434 ^a^	γ-elemene	6432312	7.04
26.242	1434	1437 ^a^	α-guaiene	5317844	0.23
26.375	1437	1439 ^a^	aromadendrene	91354	0.19
26.483	1439	1442 ^a^	guaia-6,9-diene	6427475	0.12
26.883	1449	1448 ^a^	*cis*-muurola-3,5-diene	51351708	1.4
27.075	1453	1452	α-humulene	5281520	0.86
27.408	1457	1458 ^a^	alloaromadendrene	10899740	0.29
27.608	1459	1461 ^a^	*cis*-cadina-1(6),4-diene	6431126	0.35
27.788	1466	1464 ^a^	9-*epi-(E*)-caryophyllene	6429274	0.09
27.892	1471	1475 ^a^	γ-gurjunene	90805	0.49
27.925	1475	1475 ^a^	γ-muurolene	12313020	0.7
28.325	1482	1480 ^a^	germacrene D	5373727	7.61
28.55	1488	1489 ^a^	β-selinene	442393	1.61
28.642	1490	1493 ^a^	*trans*-muurola-4(14),5-diene	91747125	0.63
28.892	1496	1499 ^a^	curzerene	572766	7.1
28.992	1498	1500 ^a^	α-muurolene	12306047	1.2
29.125	1501	1495 ^a^	γ-amorphene	12313019	0.48
29.467	1506	1508 ^a^	germacrene A	9548705	0.02
29.525	1511	1513 ^a^	γ-cadinene	92313	0.39
29.758	1516	1522 ^a^	δ-cadinene	12306054	1.86
29.925	1521	1528 ^a^	zonarene	6428488	0.48
30.317	1530	1533 ^a^	*trans*-cadina-1,4-diene	91746579	0.45
30.492	1534	1531 ^b^	selina-4(14),7(11)-diene	10655819	1.04
30.667	1539	1545 ^a^	selina-3,7(11)-diene	522296	0.25
31.375	1556	1559 ^a^	germacrene B	5281519	1.88
32.308	1582	1589 ^a^	allo-hedycaryol	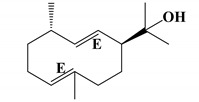 [46]	0.51
32.445	1592	1589 ^a^	*cis*-β-elemenone	519762	1.43
33.258	1602	1602 ^a^	*trans*-β-elemenone	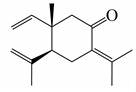 [47]	11.78
34.492	1633	1627 ^a^	cubenol<1-epi->	91753500	0.97
34.583	1643	1645 ^a^	cubenol	1770062	1.15
35.867	1661	1657 ^a^	atractylone	3080635	18.65
36.85	1694	1693 ^a^	germacrone	5317571	5.26
38.375	1739	1740 ^a^	mint sulfide	14564587	0.05
Monoterpene Hydrocarbons					1.35
Sesquiterpene Hydrocarbons					47.84
Oxygenated Sesquiterpenes					46.85
Others					0.43
**Total**					**96.47**

RI_C_: Calculated Retention Index; RI_L_: Literature Retention Index. ^a^ Adams [46]; ^b^ Nist [47]; Rt: Retention time.

**Table 2:** Antimicrobial activity of *Siparuna guianensis* leaf essential oil. Negative values (−) mean no microbial growth whereas positive values (+) mean there was microbial growth under the tested concentration.

Sample/ Dilution (µL/mL)		A	B	C	d
**1**	**500**	-	-	MIC	-
2	**250**	-	MIC	+	-
3	**125**	MIC	+	+	MIC
4	**62.5**	+	+	+	+
5	**30.625**	+	+	+	+
6	**15.3**	+	+	+	+
7	**7.6**	+	+	+	+
8	**3.8**	+	+	+	+
9	**1.9**	+	+	+	+
10	**0.95**	+	+	+	+
Mean halo, 10 µL, N = 3		11 ± 0.12	12 ± 0.57	11 ± 0,31	12.5 ± 0,98
Control		22.5 ± 0.32	28.10 ± 0.13	15.25 ± 0.58	19.42 ± 1.22

* (A) Streptococcus mutans (ATCC 3440), (B) Enterococcus faecalis (ATCC 4083); (C) Escherichia coli (ATCC 25922); (D) Candida albicans (ATCC- 10231). Inhibition halos (mm).

**Table 3:** Docking score results.

Targets	MolDock Score
*C. albicans*	−71.43
*E. coli*	−87.24
*E. faecalis*	−80.46
*S. mutans*	−65.18

**Table 4:** Energy components and values of binding affinities. All values are in kcal/mol.

Targets	ΔE_vdW_	ΔE_ele_	ΔG_GB_	ΔG_NP_	ΔG_MM-GBSA_
*C. albicans*	−22.28	−5.51	13.74	−13.11	−25.16
*E. coli*	−25.54	−6.88	15.96	−9.87	−26.33
*E. faecalis*	−19.56	−5.02	8.96	−8.22	−23.84
*S. mutans*	−24.35	−3.74	9.75	−9.13	−27.47
